# Fungal Diversity and Interactions in the Nasal and Oral Cavities of Individuals with Allergic Rhinitis, Asthma and Healthy Controls

**DOI:** 10.3390/microorganisms13061204

**Published:** 2025-05-25

**Authors:** Marcos Pérez-Losada

**Affiliations:** Computational Biology Institute, Department of Biostatistics and Bioinformatics, Milken Institute School of Public Health, The George Washington University, Washington, DC 20052-0066, USA; mlosada@gwu.edu

**Keywords:** allergic rhinitis, asthma, *ITS*, keystone taxa, mycobiome, networks, rhinitis

## Abstract

Allergic rhinitis and asthma are common chronic airway diseases that present significant public health challenges. Previous research has shown how the nasal and oral mycobiomes influence the onset, progression and severity of these two conditions, but no study so far has directly compared those mycobiomes within the same cohort during health and disease. To address this gap, I analyzed next-generation fungal *ITS* sequence data from 349 participants, including individuals with allergic rhinitis, asthma, and healthy controls. The nasal and oral mycobiomes showed a great overlap in composition but differed significantly (*p* < 0.04) in the relative abundance of several dominant genera. Moreover, only 18.6% of the fungal amplicon variants were shared among cavities. Microbial alpha-diversity was significantly higher (*p* < 0.05) in the nasal cavity, while beta-diversity varied significantly (*p* < 0.045) across all indices and clinical groups. Fungal networks were largely fragmented and showed relatively low ecological niche specialization, which contrasts with a previous study of bacteriomes from the same cohort. These networks also differed in structure, complexity and keystone nodes across clinical phenotypes. Overall, these findings highlight that the nasal and oral mycobiomes play distinct yet interconnected roles in allergic rhinitis and asthma.

## 1. Introduction

Allergic rhinitis and asthma are among the most common chronic airway diseases in developed countries, posing a significant public health burden [1,2,3]. An estimated 400 million people worldwide suffer from allergic rhinitis [4], while asthma affects over 300 million individuals and is responsible for more than 495,000 deaths annually [5,6,7,8].

Allergic rhinitis is an inflammatory condition of the nasal mucosa, characterized by sneezing, congestion, itching and rhinorrhea [9,10,11,12]. Similarly, asthma involves airway inflammation, obstruction, and mucus production [7,13,14]. These conditions frequently coexist [15,16,17,18,19], with approximately 40% of individuals with allergic rhinitis also having asthma, and up to 80% of asthma patients experiencing symptoms of allergic rhinitis [20]. This strong association supports the concept of *united airway disease*, in which both conditions share pathophysiological, epidemiological and clinical links [15,21,22,23,24].

Despite growing interest in the role of fungi in airway diseases, few studies have used next-generation sequencing (NGS) to analyze the upper airway mycobiome in individuals with rhinitis or asthma. Existing research suggests that fungal communities contribute to the onset, severity and progression of both conditions [25,26,27]. However, those studies have focused on a single niche, despite evidence that respiratory disease outcomes may be better understood by examining microbial interactions across multiple body sites [28,29,30,31,32,33,34]. Investigating cross-niche interactions could deepen our understanding of airway inflammatory diseases, support the development of holistic hypotheses on their progression, and inform more integrated diagnostic and treatment strategies [29,32,34,35,36].

In this study, I analyzed internal transcribed spacer (*ITS)* NGS data from the nasal and oral mycobiomes of 349 individuals with allergic rhinitis (with and without asthma comorbidity), asthma and healthy controls. I compared fungal taxonomic diversity and cross-cavity community interactions to explore how microbial dynamics differ between health and respiratory disease states.

## 2. Materials and Methods

### 2.1. Cohort

All participants in this study were enrolled in the ASMAPORT Project (PTDC/SAU-INF/27953/2017). This study was approved by the “Comissão de Ética para a Saúde” (Parecer_58-17, 17 March 2017) of the Centro Hospitalar Universitário São João, Facultade de Medicina (Porto, Portugal). ASMAPORT was a cross-sectional study of adults and young people from northern Portugal created in 2018 to investigate host–microbe interactions during allergic rhinitis and asthma. Further details are provided in Pérez-Losada et al. [26,27].

### 2.2. Sample Preparation and Amplicon Sequencing

Here I have included a short description of the molecular procedures performed to characterize the fugal communities of the upper airways; further details are provided in [26,27]. A total of 349 children and adults (12.7 ± 5.5 years of age and 54.2% female) participated in this study. They were classified into four clinical groups or phenotypes: allergic rhinitis (AR = 47 individuals), allergic rhinitis with asthma comorbidity (ARAS = 161), asthma (AS = 12) and healthy controls (HC = 129). Nasal and oral samples were collected by swabbing the right and left nostrils and left and right mouth cheeks, respectively, during 30 s with a cotton swab. A total of 698 samples were processed.

Total DNA was extracted using the ZymoBIOMICS™ DNA Miniprep Kit D4300 (Zymo Research Corp, Irvine, CA, USA). DNA extractions were prepared for sequencing using the Schloss’ MiSeq_WetLab_SOP protocol (09.2015) [37]. Each DNA sample, negative controls and mock samples were amplified for regions of the internal transcribed spacers (*ITS*) 1 and 2 (~230 bp) following the Earth Microbiome Project’s protocols [38]. Libraries were then sequenced in a single run of the Illumina MiSeq sequencing platform at the University of Michigan Medical School. Sequence files and associated metadata and BioSample attributes have been deposited in the NCBI (PRJNA1107919).

### 2.3. Mycobiome Analyses

*ITS* amplicon sequence variants (ASV) in each sample were inferred using dada2 version 1.18 [39]. Reads were filtered using standard default dada2 parameters. Forward and reverse reads were merged and chimeras identified. Taxonomic assignment was performed against the UNITE v9.0 2023-07-18 database [40] using the RDP naive Bayesian classifier [41,42]. ASV sequences were aligned in MAFFT [43] and a phylogenetic tree was inferred in FastTree [44]. The resulting ASV tables and tree were then imported into the R package phyloseq [45] for further analysis.

Samples were normalized using the negative binomial distribution as recommended by McMurdie and Holmes [46] and implemented in the Bioconductor package DESeq2 [47]. This approach simultaneously accounts for biological variability and library size differences and has increased sensitivity for group sample sizes of less than 20 participants. Taxonomic and phylogenetic alpha-diversity were estimated using Chao1 richness and Shannon, Simpson and phylogenetic (Faith’s) diversity (PD) indices. Beta-diversity was estimated using phylogenetic Unifrac (unweighted and weighted) distances, and dissimilarity between samples was explored using principal coordinates analysis (PCoA).

Significant differences in alpha-diversity indices and microbial abundances (phyla and genera) between nasal and oral mycobiomes in each clinical group (AR, ARAS, AS and HC) were assessed using linear models and linear mixed-effects models to account for the non-independence of subjects (random effects)—lmer4 R package [48]. Beta-diversity indices were compared using permutational multivariate analysis of variance (adonis) as implemented in the R package vegan [49]. The Benjamini–Hochberg method (alpha = 0.05) was applied to correct for multiple hypotheses testing [50,51]. All these analyses were performed in R 4.2.3 [52] and RStudio 2024.12.1 [53].

Fungal interactions were inferred for each naso–oral mycobiome (AR, ARAS, AS and HC). First ASVs were classified as nasal or oral using the niche indicator species analysis in the R package indicspec (function multipatt) [54]. This function calculates an indicator value for each ASV at each niche (mouth and nose), taking its total abundance per niche into account. An ASV was deemed a niche indicator only when it had a *p*-value < 0.05 for one specific niche. Fungal networks were then constructed using the SPIEC-EASI (SParse InversE Covariance Estimation for Ecological Association Inference) R package [55]. To avoid overestimation of the impact of very rare taxa on the overall network structure [29], only the 200 most prevalent ASVs in each group except for AS (100 ASVs) were included in the analysis (sample prevalence < 5% in all groups). Meinshausen–Buhlmann estimation with nlambda = 20, lambda.min.ratio = 0.01 and rep.num = 50 was applied [55]. This method uses conditional independence rather than correlation, hence making it less likely to detect spurious connections between taxa. Data were centered log-ratio transformed. Keystone nodes (key taxa players in the network) were selected using three criteria: degree of centrality (nodes with the highest number of connections), betweenness centrality (nodes that frequently serve as bridges in the network) and closeness centrality (nodes that can quickly reach other nodes). Only nodes in the top 95% percentile for those three indices were considered keystone taxa. The following properties were also estimated for each network: node degree (number of edges a node has with other nodes within the network), neighborhood connectivity (degree of interconnectivity among immediate neighbors in the network) and cohesion (how tightly linked are the nodes of a network).

## 3. Results

I analyzed a total of 698 nasal and oral swabs from 349 participants grouped into four phenotypes: AR = 47, ARAS = 161, AS = 12 and HC = 129 (see [26,27] for more details). After excluding singletons, the nasal–oral mycobiomes comprised 14,505,917 clean reads, ranging from 1014 to 223,989 sequences per sample (mean = 20,782.1), and 10,288 ASVs. In previous studies [26,27] I described and compared the mycobiomes of those same four clinical phenotypes within each cavity (nose and mouth) using *ITS* sequence data; here I have combined those genomic and clinical datasets to assess similarities and differences in fungal composition, structure and interactions between cavities for each clinical group (AR, ARAS, AS and HC).

### 3.1. The Nasal and Oral Mycobiomes Differ in Taxonomic Composition and Diversity

**Figure 1:**
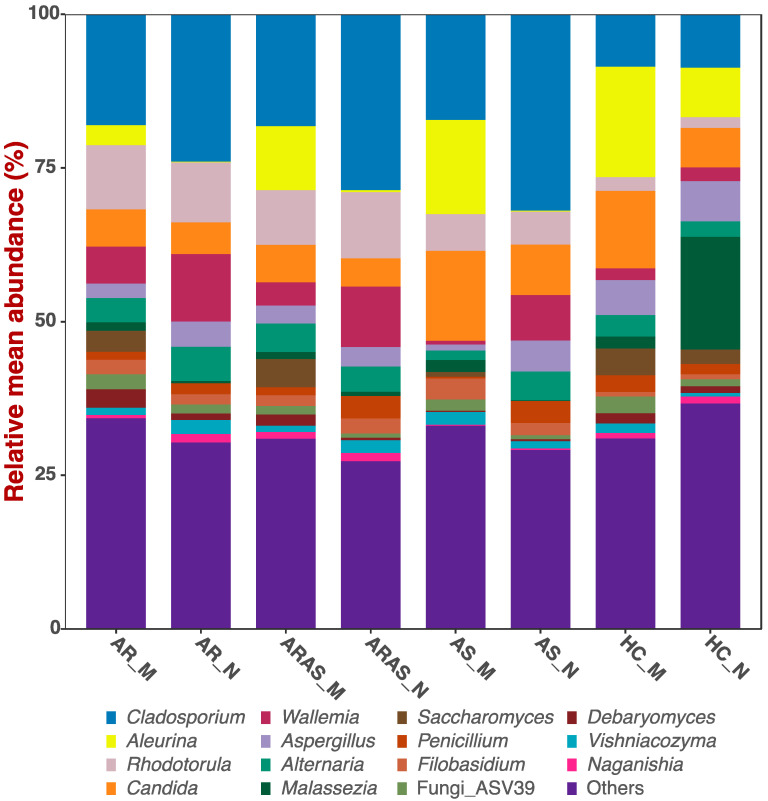
Bar plots of relative mean abundances of the top fungal genera in the mouth (M) and nose (N) of participants with allergic rhinitis (AR), allergic rhinitis with asthma comorbidity (ARAS), asthma (AS) and healthy controls (HC).

The nasal and oral mycobiomes shared the same dominant phyla and genera, but in different proportions (Table S1 and Figure 1). More information about their unique composition can be found in [26,27]. The phyla Ascomycota and Basidiomycota accounted for most of the reads in the nasal and oral mycobiomes; Ascomycota only varied significantly (*p* = 0.0002) between nose and mouth in the HC group, while Basidiomycota (*p* ≤ 0.0353) did it in the ARAS and HC groups (Table S1). Similarly, nine (*Malassezia, Cladosporium, Penicillium, Aspergillus, Candida, Aleurina, Debaryomyces, Wallemia* and *Saccharomyces*) of the fifteen dominant fungal genera showed significant differences (≤0.0387) in mean relative abundance across phenotypes (Table S1). *Cladosporium* varied significantly across all the disease phenotypes, *Penicillium* in ARAS and AS, *Aleurina* in ARAS and HC and *Debaryomyces, Wallemia* and *Saccharomyces* in AR and ARAS. The other three genera (*Malassezia, Aspergillus* and *Candida*) only varied significantly in one particular phenotype.

As a whole (all phenotypes combined), the nasal and oral mycobiomes had 4025 and 4653 unique ASVs, respectively, and shared 1610 (18.6%) ASVs. Then, within each clinical group, AR, ARAS, AS and HC shared eleven, one hundred and ninety-three, three and ninety-five ASVs, respectively, between cavities (Figure S1).

**Figure 2:**
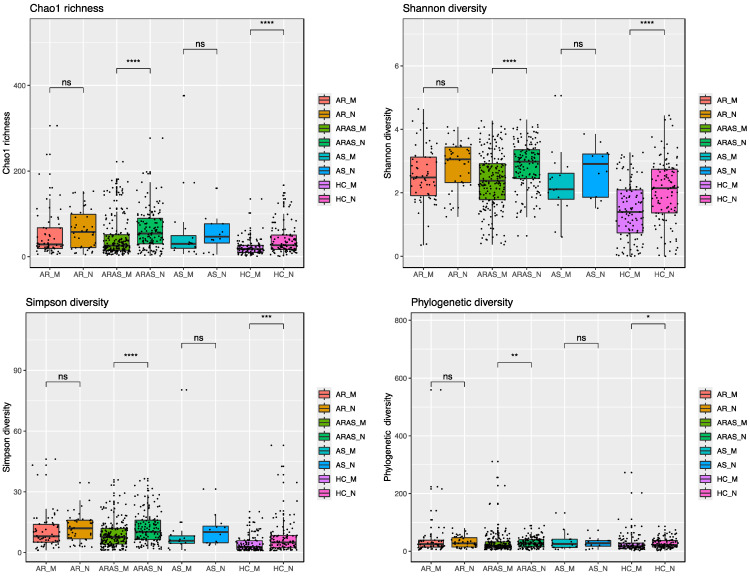
Alpha-diversity estimates in mycobiomes from the mouth (M) and nose (N) of participants with allergic rhinitis (AR), allergic rhinitis with asthma comorbidity (ARAS), asthma (AS) and healthy controls (HC). Statistical significance is indicated: * = *p* ≤ 0.05; ** = *p* ≤ 0.01; *** = *p* ≤ 0.001; **** = *p* ≤ 0.0001; ns = not significant.

Alpha-diversity indices [Chao1, Shannon, Simpson and phylogenetic diversity (PD)] of community richness and evenness varied between nasal and oral mycobiomes for each phenotype (Figure 2 and Table S2). The nasal mycobiomes showed higher diversity than the oral mycobiomes for all the indices and phenotypes except for PD and Chao1 in AS. All of the alpha-diversity pairwise comparisons between cavities across phenotypes resulted significant (*p* < 0.01), except for Chao1 in AR and AS, and PD in AS.

**Figure 3:**
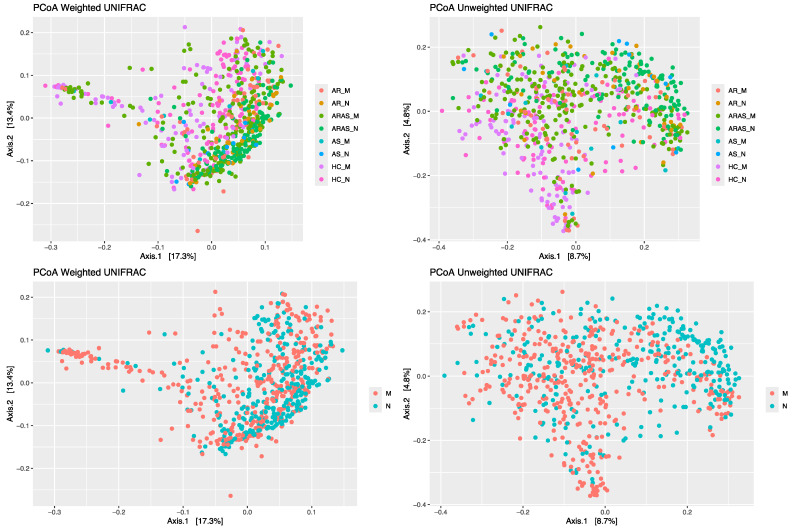
Principal coordinates analysis (PCoA) plots of beta-diversity Unifrac distances in mycobiomes from the mouth (M) and nose (N) of participants with allergic rhinitis (AR), allergic rhinitis with asthma comorbidity (ARAS), asthma (AS) and healthy controls (HC).

To characterize the structure of the fungal communities (beta-diversity), I applied principal coordinates analysis (PCoAs) to Unifrac (unweighted and weighted) distance matrices. The PCoAs of the samples grouped either by cavity alone or by cavity and clinical phenotype (Figure 3) separated the nasal and oral mycobiomes, although not completely. Axes one and two in the PCoA of Unifrac weighted distances explained 17.3% and 13.4% of the variance, respectively, while the same axes in the PCoA of Unifrac unweighted distances explained 8.7% and 4.8% of the variance, respectively. Group segregation was confirmed by the adonis test, which revealed significant differences (0.0433 ≤ *p* < 0.0001) in beta-diversity for all indices and pairwise comparisons performed.

### 3.2. Fungal Interactions in the Nasal and Oral Cavities

**Figure 4:**
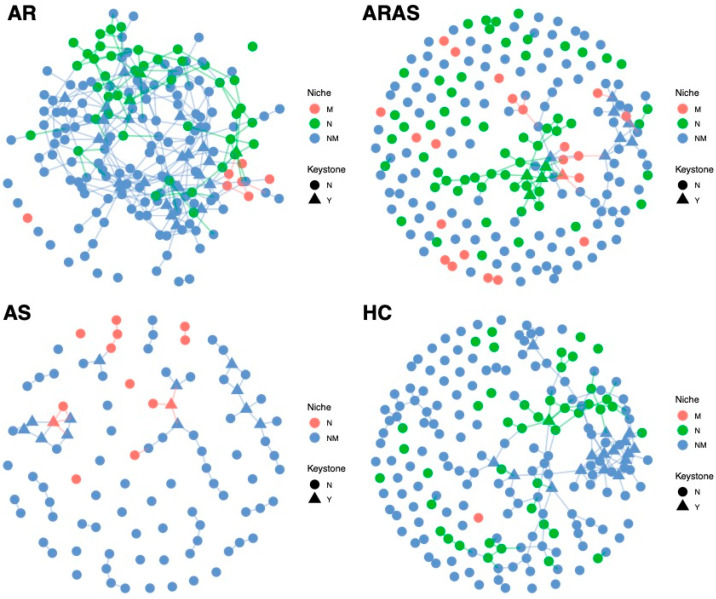
Co-occurrence networks of naso–oral mycobiomes from participants with allergic rhinitis (AR), allergic rhinitis with asthma comorbidity (ARAS), asthma (AS) and healthy controls (HC). All nodes (ASVs) in the networks were assigned to a cavity (M = mouth, N = nose, NM = mixed or undetermined).

I inferred fungal interactions in the naso–oral mycobiomes of each phenotypic group (AR, ARAS, AS and HT) using SPIEC-EASI analysis (Figure 4). All nodes (ASVs) in each network were statistically assigned to a cavity (M = mouth, N = nose or NM = undetermined or mixed), as indicated by niche indicator analysis. ASVs in N and M represent specialists strongly associated to each cavity, while ASVs in NM represent generalists occurring in both cavities (low-prevalence ASVs were filtered out) (Table S3).

All naso–oral networks looked different in their structure, complexity and keystone nodes (Table S3). The AR network included 200 nodes (M = 7, N = 44, NM = 149) and 322 edges, with 14.0% of them connecting intra-niche ASVs (N-N or M-M) and 86% connecting inter-niche ASVs (N-M, N-NM, M-NM and NM-NM). The AR network showed a mean node degree of 3.22, a mean neighborhood connectivity of 3.92, a mean cohesion of 0.11. It included 25 keystone taxa (M = 0, N = 3 and NM = 22) belonging to the genera *Bullera, Cladosporium, Crustomyces, Debaryomyces, Exophiala, Fusarium, Ganoderma, Malassezia, Mycosphaerella,* Mycosphaerellaceae_sp, *Papiliotrema, Peniophora, Phaeophlebiopsis, Phlebia, Phlebiopsis, Rhodotorula, Sidera, Sistotrema, Skeletocutis, Stereum, Symmetrospora* and *Vishniacozyma*.

The ARAS network included 200 nodes (M = 22, N = 61, NM = 117) and 117 edges with 35.9% of them connecting intra-niche ASVs (64.1% inter-niche) and many unconnected nodes. It showed a mean node degree of 1.17, a mean neighborhood connectivity of 1.61 and a mean cohesion of 0.05. This network included 12 keystone taxa (M = 1, N = 4 and NM = 7) of the genera *Cladosporium, Debaryomyces, Laetiporus, Mycoacia, Phlebia, Sidera, Sistotrema* and *Sterigmatomyces*.

The AS network included 100 nodes (M = 0, N = 13 and NM = 87) and 66 edges with 7.6% of them connecting intra-niche ASVs (92.4% inter-niche). It showed a mean node degree of 1.32, a mean neighborhood connectivity of 1.58 and a mean cohesion of 0.04. The AS network included 14 keystone taxa (M = 0, N = 2 and NM = 12) of the genera *Acremonium*, *Bjerkandera*, *Filobasidium*, *Itersonilia*, *Meyerozyma*, *Penicillium*, *Phaeosphaeria*, *Phanerochaete*, *Pseudobensingtonia*, *Sporobolomyces*, *Stereum* and *Vishniacozyma.*

Finally, the HC network included 200 nodes (M = 1, N = 43 and NM = 156) and 192 edges with 11.5% of them connecting intra-niche ASVs (88.5% inter-niche). It showed a mean node degree of 1.92, a mean neighborhood connectivity of 2.63 and a mean cohesion of 0.09. The HC network included 17 keystone taxa (M = 0, N = 1 and NM = 16) of the genera *Agaricomycetes_sp*, *Aspergillus*, *Athelia*, *Chytridiomycota_gen_Incertae_sedis*, *Cladosporium*, *Constantinomyces*, *Penicillium*, *Rigidoporus*, *Saccharomyces*, *Toxicocladosporium* and *Wallemia*.

Four keystone ASVs were shared between AR and ARAS, one between AR and AS, and two between AS and HC (Table S3). Similarly, five keystone fungal genera were shared between AR and ARAS, two between AR and AS, one between AS and HC, and one among AR, ARAS and HC (Table S3).

## 4. Discussion

Allergic rhinitis and asthma are significant healthcare burdens, causing widespread distress globally [1,2,3]. The nasal and oral mycobiome have been closely linked to the onset, progression and severity of both conditions [25,26,27]. However, no studies so far have directly compared these two microbial communities within the same cohort to evaluate their similarities, differences and potential interactions during rhinitis or asthma. To bridge this gap, I analyzed *ITS* sequencing data from 349 individuals (698 nasal and oral samples), including those with allergic rhinitis (with and without comorbid asthma), asthma and healthy controls.

The nasal and oral mycobiomes overlapped in phyla and genera composition across all studied phenotypes (AR, ARAS, AS and HC) (Table S1 and Figure 1). All these fungal taxa are natural residents of the two cavities, including some opportunistic pathogens (e.g., *Malassezia*, *Alternaria*, *Aspergillus*, *Candida* and *Penicillium*) [56,57,58]. Fungal abundances varied more greatly between cavities at the genus level than at the phylum level, with 2–3 dominant genera varying significantly in AS and HC and 5–6 in AR and ARAS (Table S1). The nasal mycobiomes showed higher richness and evenness than the oral mycobiomes for most alpha-diversity indices and phenotypes (Figure 2 and Table S2), with about half of the comparisons resulting significant (*p* < 0.01). Nasal and oral fungal communities were also differently structured across cavities and clinical phenotypes for all beta-diversity indices compared (0.0433 ≤ *p* < 0.0001), with the former being the major driver of divergence (Figure 3).

As indicated above, we are still lacking studies comparing the composition and diversity of the nasal and oral mycobiomes in rhinitis or asthma, but previous analyses in healthy individuals have revealed shared membership but significant differences in relative proportions between both cavities [59,60,61,62], as seen in this study. Such taxonomic differences, as those seen in diversity, can be attributed to several key factors differentiating the nasal and oral mucosal environments, including oral exposure to food and beverage intake, variation in environmental conditions like pH (the mouth tends to be more neutral, while the nasal cavity is slightly more acidic); moisture (the mouth has abundant saliva, while the nose has mucus) or oxygen availability (the nasal passages are more directly exposed to ambient air compared to the relatively more enclosed and moist oral cavity); host immune factors (the saliva, for example, contains antimicrobial peptides; immunoglobulins and enzymes like lysozyme that regulate fungal populations differently than the mucociliary defense system in the airways; similarly, the nasal mucus has also unique antimicrobial peptides); intra- and inter-domain microbial interactions (the bacterial communities also differ between the mouth and nose, influencing fungal populations through competition or cooperation); biofilms (like the dental plaque, which provides a unique structure that supports fungi differently than the loosely attached microbiota in the nasal cavity); anatomical barriers and mucociliary clearance mechanisms (lacking in the oral cavity) [59,60,61,62,63,64,65].

Co-occurrence networks showed distinct patterns of fungal interactions in each phenotype (Figure 4 and Table S3). The AR network showed the highest connectivity (i.e., complexity), while the other three networks shower a greater proportion of unconnected nodes (i.e., fragmentation). This agrees with a former network analysis of the oral mycobiome, which also shown more complex interactions in the AR group [27]. The AS network showed the lowest proportion (13%) of specialists (N or M), while the ARAS network showed the highest proportion (41.5%) of specialists. Higher proportions of NM taxa suggest that AR, AS and HC networks are less stable and more transient. Concomitantly, the ARAS network also showed the highest proportion (36%) of intra-niche node interactions, while the other three naso–oral networks showed lower values (8% to 14%). Most of the keystone nodes across the networks (58.3 to 94.1%) also inhabited both cavities (NM). All this evidence, thus, suggests that the fungal interactome in the naso–oral region is dominated by generalists. This remarkably contrasts with a previous study of the naso–oral bacteriomes in this same cohort [34], where network membership included more specialists (up to 99.4% in the HC network) and lower fragmentation, highlighting differences between the fungal and bacterial interactomes.

Previous studies of the oral, nasal and nasopharyngeal bacteriome during respiratory infection have also suggested that loss of ecological specialization (or translocation) may lead to respiratory disease [29,66,67,68]. Similarly, network fragmentation, which is indicative of potential dysbiosis and loss of stability, could increase susceptibility to airway disease [29,34]. The co-occurrence analyses here do not seem to show clear trends, since healthy individuals showed similar or lower levels of fungal network specialization and fragmentation than AR and ARAS individuals (Figure 4 and Table S3). Although it is possible that those factors are playing differential roles in these communities (HC included less pathogenic keystone taxa, hence less dysbiotic interactions—see below), further research is needed to validate this hypothesis.

Taxa inhabiting both cavities (i.e., generalists) seem to play an important role in the fungal interactome. These findings highlight the distinct yet interconnected roles of the naso–oral mycobiome in allergic rhinitis and asthma. Moreover, they reinforce the idea that, despite their shared pathophysiological and clinical features under the united airway disease concept [15]), allergic rhinitis and asthma may represent distinct conditions, as supported by this and other omics studies [26,27,34,69,70,71,72,73].

Twelve to twenty-five keystone nodes (key taxa influencing community structure, stability and function) were detected across the networks (Table S3). Several belonged to genera considered opportunistic pathogens (*Acremonium*, *Fusarium*, *Exophiala*, *Malassezia*, *Meyerozyma* or *Rhodotorula*) and were only found in the disease phenotypes. Nonetheless, some pathogenic genera (*Cladosporium, Penicillium* or *Aspergillus*) were also found in the healthy group, which included more commensal taxa.

Some keystone ASVs of the genera Aspergillus, Cladosporium, Debaryomyces, Filobasidium, Malassezia, Penicillium, Rhodotorula, Saccharomyces, Vishniacozyma and Wallemia showed a relative high abundance in the nasal or oral cavities; however, most of them (Table S3) showed low abundances, despite being highly connected. This aligns with the “rare taxa” hypothesis, which suggests that a species’ abundance is not always the best indicator of its significance within a microbial community [74,75]. Adopting a system-centric approach to studying the airway mycobiome may offer deeper insights into the roles of lesser-known microbes as disease indicators and potential therapeutic targets [29,34,76,77,78,79]. Further research is needed to elucidate their contribution to the pathogenesis of respiratory illnesses [71,72,73,79,80,81,82].

This study has some limitations. *ITS* amplicon sequence data has limited resolution, hence fungal taxa could not be widely identified at the species level. *ITS* sequencing may experience PCR biases [83,84]. This is a cross-sectional study, so it does not account for potential fungal variation over time or the impact of confounding factors (antibiotic or probiotic use, clinical severity or allergens). Similarly, it allows to infer significant associations across the compared clinical groups but not causality. The potential pathological effect of pathogenic fungi in patients with allergic rhinitis or asthma is beyond the scope of this study, and further research is needed to explore that. Finally, this study focuses on a Portuguese cohort, hence new cohorts need to be characterized to validate all the findings presented here.

## 5. Conclusions

I analyzed the nasal and oral mycobiomes of a cohort of 349 individuals, including those with allergic rhinitis (with and without comorbid asthma), asthma and healthy controls. The fungal communities exhibited significant differences in taxonomic composition, diversity and structural organization between cavities across all clinical groups. Additionally, fungal networks differed notably in connectivity and fragmentation and keystone taxa, with multiple keystone species of varying relative abundance identified in each network.

## Data Availability

Sequence files and associated metadata and BioSample attributes have been deposited in the NCBI (PRJNA1107919).

## Supplementary Materials

The following supporting information can be downloaded at: https://www.mdpi.com/article/10.3390/microorganisms13061204/s1. Table S1: Mean relative proportions and statistical significance of pairwise comparisons of mycobiomes from the mouth (M) and nose (N) of participants with allergic rhinitis (AR), allergic rhinitis with asthma comorbidity (ARAS), asthma (AS) and healthy controls (HC). Table S2: Alpha-diversity estimates (Chao1 richness, Shannon, Simpson and phylogenetic diversity indices) of mycobiomes from the mouth (M) and nose (N) of participants with allergic rhinitis (AR), allergic rhinitis with asthma comorbidity (ARAS), asthma (AS) and healthy controls (HC). Table S3: Amplicon sequence variants (ASVs) included in the co-occurrence networks (nodes) of the naso–oral mycobiomes in Figure 4. Niche assignment (N = nose, M = mouth, NM = undetermined or mixed), keystone status and taxonomic identification are provided for all ASVs. Figure S1: UpSet plot of amplicon sequence variants (ASVs) of mycobiomes from the mouth (M) and nose (N) of participants with allergic rhinitis (AR), allergic rhinitis with asthma comorbidity (ARAS), asthma (AS) and healthy controls (HC).
