# Supplementary figures and images for: Fungal Diversity and Interactions in the Nasal and Oral Cavities of Individuals with Allergic Rhinitis, Asthma and Healthy Controls
Source: Microorganisms. 2025 May 25;13(6):1204. doi: 10.3390/microorganisms13061204 (PMC12195179; doi:10.3390/microorganisms13061204)

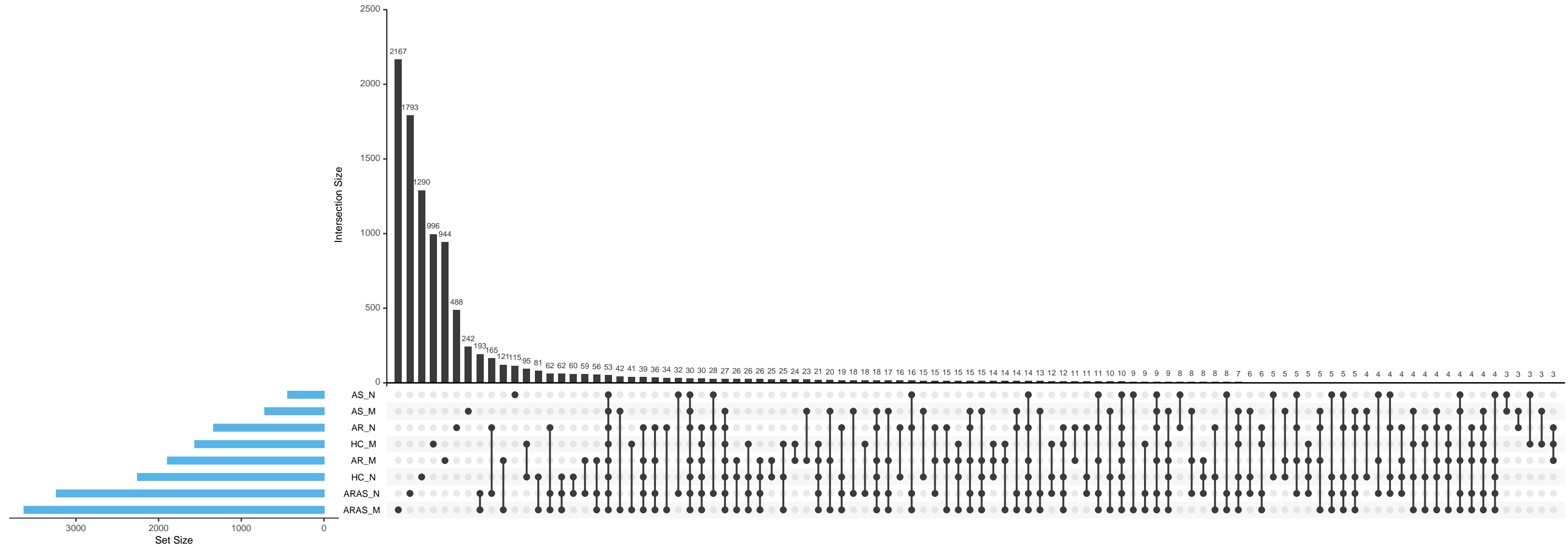

Supplement: Supplementary file 1 [file microorganisms-13-01204-s001.zip › microorganisms-3584850-supplementary.pdf]
